# Predictive Performances of Blood-Count-Derived Inflammatory Markers for Liver Fibrosis Severity in Psoriasis Vulgaris

**DOI:** 10.3390/ijms242316898

**Published:** 2023-11-29

**Authors:** Oana Mirela Tiucă, Silviu Horia Morariu, Claudia Raluca Mariean, Robert Aurelian Tiucă, Alin Codrut Nicolescu, Ovidiu Simion Cotoi

**Affiliations:** 1 Doctoral School of Medicine and Pharmacy, George Emil Palade University of Medicine, Pharmacy, Science, and Technology of Targu Mures, 540142 Targu Mures, Romania; 2 Dermatology Department, George Emil Palade University of Medicine, Pharmacy, Science, and Technology of Targu Mures, 540142 Targu Mures, Romania; 3 Dermatology Clinic, Mures Clinical County Hospital, 540342 Targu Mures, Romania; 4 Pathophysiology Department, George Emil Palade University of Medicine, Pharmacy, Science, and Technology of Targu Mures, 540142 Targu Mures, Romania; 5 Endocrinology Department, George Emil Palade University of Medicine, Pharmacy, Science, and Technology of Targu Mures, 540142 Targu Mures, Romania; 6 Endocrinology Department, Mures Clinical County Hospital, 540139 Targu Mures, Romania; 7 Agrippa Ionescu Emergency Clinical Hospital, 011773 Bucharest, Romania; 8 Pathology Department, Mures Clinical County Hospital, 540011 Targu Mures, Romania

**Keywords:** psoriasis, inflammation, fibrosis, non-invasive, risk-assessment

## Abstract

Psoriasis is an immune-mediated, chronic disorder that significantly alters patients’ quality of life and predisposes them to a higher risk of comorbidities, including liver fibrosis. Various non-invasive tests (NITs) have been validated to assess liver fibrosis severity, while blood-count-derived inflammatory markers have been proven to be reliable in reflecting inflammatory status in psoriatic disease. The fibrosis-4 (FIB-4) index became part of the newest guideline for monitoring psoriasis patients undergoing systemic treatment. Patients with psoriasis vulgaris and fulfilling inclusion criteria were enrolled in this study, aiming to assess for the first time in the literature whether such inflammatory markers are useful in predicting liver fibrosis. Based on internationally validated FIB-4 index values, patients were divided into two study groups: a low risk of significant fibrosis (LR-SF) and a high risk of significant fibrosis (HR-SF). Patients from HR-SF were significantly older and had higher values of the monocyte-to-lymphocyte ratio (MLR) (*p* < 0.001), which further significantly correlated with fibrosis severity (*p* < 0.001). Platelet-to-lymphocyte ratio (PLR), systemic immune inflammation index (SII), platelet-to-white blood cell ratio (PWR), and aggregate index of systemic inflammations (AISI) significantly correlated negatively with liver fibrosis (*p* < 0.001). PWR proved to be the most reliable inflammatory predictor of fibrosis severity (AUC = 0.657). MLR, PWR, and AISI were independent inflammatory markers in multivariate analysis (*p* < 0.001), while the AST to platelet ratio index (APRI) and AST to ALT ratio (AAR) can be used as additional NITs for significant liver fibrosis (*p* < 0.001). In limited-resources settings, blood-count-derived inflammatory markers such as MLR, PWR, and AISI, respectively, and hepatic indexes APRI and AAR prove to be of particular help in predicting significant liver fibrosis.

## 1. Introduction

Psoriasis is an immune-mediated, chronic disorder that significantly alters patients’ quality of life (QoL), affecting between 1.5% and 5% of the population in developed countries [1] (recently estimated at 4.99% in Romania [2]). 

Cutaneous lesions may vary depending on the psoriasis subtype, but the most common morphology refers to well-defined erythematous and scaly plaques, reflecting skin inflammation, epidermal hyperplasia, and angiogenesis due to altered immune pathways. A continuous interaction between dendritic cells, T cells, and keratinocytes leads to increased production of pro-inflammatory molecules, promoting an increased inflammatory state, that transcends skin level and has a systemic impact.

Patients with psoriasis have a higher risk of developing cardiovascular diseases, especially hypertension [3] and atherosclerosis, metabolic disorders, inflammatory bowel disease, psychiatric disorders, and kidney disease [4]. Additionally, psoriasis is linked to major cardiovascular events, such as myocardial infarction and stroke, especially in those with severe and prolonged courses of disease.

Psoriasis patients are more likely to have liver fibrosis [5], partly due to the interleukin-17 pathway [6]. Drug-induced fibrosis, especially methotrexate-related, should also be taken into account. Even though previous guidelines recommended monitoring possible liver disease with routine blood analysis and liver biopsy [7], more recent evidence considers routine liver enzymes to be non-reliable [8] and liver biopsy to be too invasive. 

Recent advances in the field revealed that non-invasive tests (NITs) are of great help in clinical practice and can confidently rule out the presence of advanced fibrosis (AF) [9], with patients at a high risk of AF being offered further testing, and those at low risk of AF benefiting from annual re-evaluation. NITs have become an integral part of the most recent AAD guideline [10] for baseline evaluation of liver fibrosis. Patients should benefit first-hand from liver fibrosis evaluation by the means of NITs, such as Fibrosis-4 (FIB-4) index, Fibrosure, Fibrometer, or Hepascore, and if proven to be at low risk of AF, methotrexate treatment can safely be initiated. Additionally, NITs, such as AST to platelet ratio index (APRI), AST to ALT ratio (AAR), and GGT to platelet ratio index (GPR) have been described, but their usefulness has not been until now widely accepted. 

Blood-count-derived inflammatory markers, such as neutrophil-to-lymphocyte ratio (NLR), platelet-to-lymphocyte ratio (PLR), or systemic immune inflammation index (SII) have gained increased interest in recent years. They were proven to be associated with and predicted outcomes in patients with cardiovascular disease [11,12,13], tumors [14,15,16,17,18], and kidney disease [19,20]. For skin disorders, the usefulness of these markers has been assessed in erythema nodosum [21], Behcet disease [22], and sarcoidosis [23]. In psoriasis, these markers have been proven to be reliable in assessing both the disease’s presence and its severity [24,25,26]. The platelet-to-white blood cell ratio (PWR), a potential biomarker of vascular inflammation predicting acute ischemic stroke and cardiovascular risk [27,28], has never been assessed in relation to psoriasis. Moreover, the usefulness of PWR in hepatic diseases has been, until now, tested only in acute-to-chronic liver failure (ACLF) [29], HBV-positive patients [30], and pyogenic liver abscesses [31].

To the best of our knowledge, until now, no study has evaluated the reliability of blood-count-derived inflammatory markers in assessing liver fibrosis. This study aimed to establish the prognostic value of inflammatory markers in predicting liver fibrosis severity in patients with psoriasis based on an international consensus regarding NITs. 

## 2. Results

### 2.1. Patients’ Clinical Profile

A total of 359 patients diagnosed with psoriasis were included in this study. Most of them were males (n = 216), with a mean age at enrollment of 54.76 ± 16.36. Regarding psoriasis severity, 177 presented with mild disease, while 182 had moderate-to-severe psoriasis. As depicted in Table 1, 246 patients had mild fibrosis (LR-SF, median FIB-4 = 0.69; 95% CI: 0.65–0.75), while 113 presented moderate-to-severe liver fibrosis (HR-SF, median FIB-4 = 2.08; 95% CI: 1.78–2.66). Patients in the HR-SF group were significantly older (*p* < 0.001) than those in the non-AF group. Most patients in the LR-SF group had mild psoriasis (53.66%), while in the HR-SF group most patients presented with moderate-to-severe psoriasis (60.18%).

Patients in the HR-SF group had significantly higher values of ALT, AST, GGT, MLR, APRI, AAR, and GPR, while those with LR-SF presented higher levels of WBC, platelets, neutrophils, lymphocytes, PLR, SII, AISI, and PWR. No statistically significant differences were identified regarding monocyte count, NLR, d-NLR, and SIRI between the two study groups. Nevertheless, patients with HR-SF had higher NLR and d-NLR than those with LR-SF. Moreover, thrombocytopenia was more frequently encountered in HR-SF (22/113; 19%), while only two patients from LR-SF presented this blood alteration (0.008%).

### 2.2. Serological Markers and Liver Fibrosis Scores

The association of serological markers with liver fibrosis was further analyzed. As fibrosis progressed, PLR decreased. Spearman’s correlation analysis revealed (Table 2) that platelet count, WBC, neutrophil and lymphocyte count, PLR, SII, AISI, and PWR were significantly and negatively correlated with liver fibrosis. On the other hand, AST, ALT, GGT, MLR, APRI, AAR, and GPR were strongly positively correlated with liver fibrosis. No correlation was identified between NLR, d-NLR, and SIRI values and liver fibrosis.

### 2.3. Performance of Inflammatory Biomarkers for the Evaluation of Liver Fibrosis

The diagnostic performances of different markers are demonstrated in Table 3. The AUC of PLR for evaluating significant fibrosis was 0.618 (95% CI = 0.565–0.668) with a cut-off value of 94.68, while the AUC of MLR for evaluating significant fibrosis was 0.624 (95% CI = 0.571–0.674) with a cut-off of 0.26. The AUC of SII for evaluating significant fibrosis was 0.640 (95% CI = 0.588–0.690) with a cut-off at 828.77, AISI predicted significant fibrosis with an AUC of 0.607 (95% CI = 0.555–0.658) and a cut-off value of 273.09, while PWR predicted significant fibrosis with an AUC of 0.657 (95% CI = 0.606–0.706) and a threshold value of 27.59.

**Figure 1:**
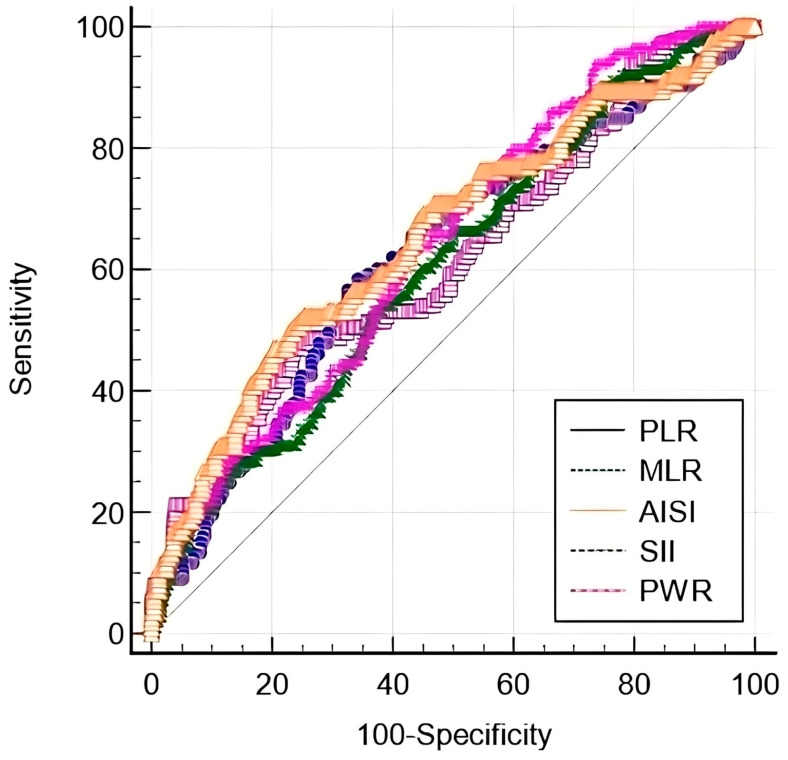
ROC comparison of PLR, MLR, AISI, SII, and PWR in predicting significant liver fibrosis.

SII had the highest sensitivity, while PLR had the highest specificity. Comparing AUCs of different serum models for predicting significant liver fibrosis, the AUC of PWR was the highest, but comparable with PLR (*p* = 0.19), MLR (*p* = 0.44), SII (*p* = 0.71), and AISI (*p* = 0.33) (Figure 1). 

### 2.4. Performance of Hepatic NITs for the Evaluation of Liver Fibrosis

APRI (r = 0.63; *p* < 0.001), AAR (r = 0.44; *p* < 0.001), and GPR (0.46; *p* < 0.001) were positively and statistically significantly correlated with liver fibrosis severity. The AUCs of APRI, AAR, and GPR for assessing liver fibrosis were 0.889 (95% CI = 0.852–0.920), 0.774 (95% CI = 0.727–0.816), and 0.786 (95% CI = 0.740–0.828), respectively, with cut-offs 0.22, 0.89, and 0.14, respectively (Table 4). 

**Figure 2:**
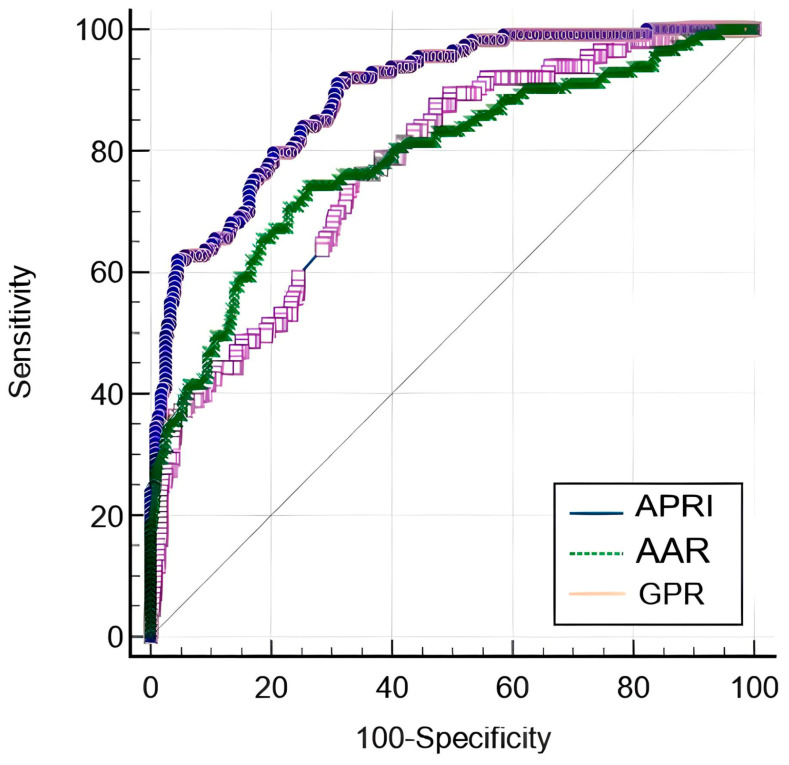
ROC comparison of APRI, AAR, and GPR for predicting significant liver fibrosis.

Out of these indexes, APRI had the highest sensitivity, while GPR had the highest specificity. The AUC of APRI was higher compared to AAR (*p* < 0.001) and GPR (*p* < 0.001), as depicted in Figure 2. 

### 2.5. The Reliability of Blood-Count-Derived Markers for Predicting Liver Fibrosis Severity

In a multivariate logistic regression model (Table 5), patients aged more than 50 years old (OR: 4.63, *p* < 0.001) and presenting with moderate-to-severe psoriasis (OR: 1.70, *p* = 0.028) were identified to have a higher risk of significant liver fibrosis. Moreover, higher levels of MLR (OR:3.51, *p* < 0.001), APRI (OR = 11.68, *p* < 0.001), and AAR (OR = 13.26, *p* < 0.001), and lower levels of AISI (OR = 0.98, *p* = 0.009) and PWR (OR = 0.94, *p* < 0.001) were independent predictors of significant liver fibrosis.

## 3. Discussion

Early detection and a proper assessment of liver inflammation and fibrosis are important not only for disease progression but also necessary when dealing with multifactorial and complex diseases such as psoriasis, which very often require systemic treatment. Additionally, psoriatic patients may require a personalized approach, taking into account associated comorbidities and data reported in the literature. 

Keratinocytes play a key role in psoriasis etiopathogenesis. By providing antimicrobial peptides like S100A7 that bind to host DNA, they initiate the stimulation of dendritic cells. Activated dendritic cells lead to increased production of proinflammatory markers such as IL-12, IL-23, IL-8, IL-17, and TNF-α [32,33,34,35,36]. Moreover, the genetic bases of psoriasis, defined by more than 50 psoriasis susceptibility loci with PSOR1 being the most important, modulate immune pathways that further increase disease susceptibility, such as the IL-23/IL-17 axis and the type I IFN pathway [37]. Nevertheless, even though these cytokines are the hallmarks of psoriasis etiopathogenesis and are proven to be reliable markers of disease progression, they are not widely used in daily clinical practice, most likely due to highly specialized techniques used for their detections and high costs. 

Blood-count-derived inflammatory markers have been reported to be reliable in cardiovascular diseases, tumors, and kidney disease. Studies referring to psoriasis tested the reliability of these markers both as diagnostic and prognostic factors and also their usefulness in assessing a patient’s response to different therapeutical means, such as conventional immunosuppressants and innovative (both biological and non-biological) drugs [38,39,40,41,42,43]. Andersen et al. [43] identified that a higher pretreatment with PLR and SII were less likely to respond to conventional systemic agents, while Asahina et al. [39] proved that NLR and PLR decrease in the same manner as C-reactive protein (CRP) in patients undergoing biological therapy, no matter the biologic agent that was used. Anti-TNF agents, such as adalimumab, infliximab, and etanercept, seem to be more effective in decreasing NLR and CRP values compared to IL inhibitors ustekinumab and secukinumab [44]. Moreover, biologics seem to decrease the proinflammatory cytokines TNF-α [45], IL-6, and IL-22 [46]. Additionally, infliximab [47] and secukinumab [48] decrease oxidative stress levels and increase total antioxidant status [47], adalimumab and etanercept increase superoxide dismutase and glutathione levels and decrease nitric oxide [49], while efalizumab [47] and ustekinumab [50] decrease malondialdehyde levels. On the other hand, methotrexate elevates malondialdehyde, caspase-3, and oxidative stress levels [51]. As such, apart from decreasing inflammatory status, biologics may have protective effects against oxidative stress, a key pathogenic factor in psoriasis development.

An increased inflammatory status in psoriatic disease is further reflected in associated comorbidities. It should be noted that patients with psoriasis present an additional comorbid risk derived from the choice of treatment. 

Psoriasis patients are prone to liver fibrosis, partly due to the interleukin-17 pathway. IL-17 signaling increases the expression of a fibrogenic cytokine, the transforming growth factor-1, and induces the production of type 1 collagen in hepatic stellate cells by activating the Stat3 pathway [6]. On the other hand, methotrexate leads to liver fibrosis by increasing extracellular adenosine in stellate cells [52], while acitretin promotes fibrogenesis by impacting the mitochondrial function of stellate cells and leading to apoptosis and necrosis of these cells. In addition, IL-22 and IL-23 seem to decrease liver fibrosis [6]. As such, screening for liver fibrosis is essential because it identifies patients at risk and guides treatment decisions.

Our study was based on FIB-4 and not other NITs due to the fact this marker was integrated into the latest guidelines [10] for assessing liver fibrosis. While in resource-limited settings, liver fibrosis scores calculated from simple laboratory values, such as the FIB-4 index, are useful for identifying patients who may need additional testing, allowing, therefore, better resource management and therapeutic decisions. Patients with an FIB-4 lower than 1.3 are considered to being of having a low risk of significant fibrosis (F0-F1) and should benefit from periodical monitoring, while those with values higher than this cut-off benefit from further testing. Additionally, a FIB-4 > 3.25 is considered to indicate significant liver fibrosis (>F2) [53,54].

Our study identified that patients with HR-SF presented with decreased values of peripheral neutrophils and lymphocytes, probably due to the migration of these cells from the blood to the liver. Patients with significant liver fibrosis lose more lymphocytes than neutrophils in their peripheral blood, as indicated by elevated NLR and d-NLR in HR-SF. On the other hand, high levels of monocytes in HR-SF can be attributed to ongoing bone marrow inflammation and monocyte mobilization to the periphery. The relationship between NLR and liver fibrosis was explored in other studies, with conflicting results [55,56]. In a study published by Kara et al. [56], NLR was not associated with the severity of liver fibrosis, while Ülger et al. [55] described that low NLR values are useful in predicting advanced liver fibrosis in HCV-positive patients. In our study, NLR and d-NLR did not exhibit differences between LR-SF and HR-SF. MLR significantly differed between study groups and correlated positively with fibrosis severity, while PLR, SII, AISI, and PWR negatively correlated with liver fibrosis. Other studies reported that PLR is useful in evaluating liver fibrosis and inflammation [57] and could perform comparably to FIB-F [58]. All markers were good indicators of liver fibrosis, with an AUC > 0.60. Our study reports for the first time in the literature, PWR as an inflammatory marker in psoriasis and its usefulness in predicting liver fibrosis severity. It had the highest performance to assess HR-SF with an AUC of 0.657. However, after running a multivariate regression model, only MLR, PWR, and AISI proved to be significant independent predictors of liver fibrosis. Thrombocytopenia was more frequently encountered in HR-SF, most likely due to decreased thrombopoietin levels in advanced liver disease and direct bone marrow suppression [59].

Significant differences were also noted between the two study groups regarding AST, ALT, and GGT. However, these markers should not be individually used to assess liver fibrosis since they can easily be influenced by various factors, such as diet, living habits, and metabolic status. We also evaluated combined parameters such as AAR, APRI, and GPR, which were proved to indicate the presence and severity of liver fibrosis in chronic hepatitis C [60,61,62,63]. AAR and GPR displayed good predictive value (AUC > 0.60), while APRI, which had the highest performance to stage HR-SF with an AUC of 0.889 and was superior to AAR and GPR, had a very good predictive value. Our analysis also identified that APRI and AAR can be used as prognostic factors of liver fibrosis. Additionally, APRI and AAR proved to be prognostic factors of HR-SF.

Disease severity, male sex, and age > 50 years old were also identified as predictors of HR-SF. This indicates the need for additional screening for these patients and exemplifies once more the direct link between psoriatic disease and systemic comorbidities. 

The main limitation of this study lies in its single-center retrospective character. Alcohol intake was evaluated based on clinical records and patients with psoriasis vulgaris who reported a daily intake of alcohol were excluded; no systematically quantified level of alcohol intake was available due to the retrospective nature of data collection. Psoriasis severity was assessed using only the BSA score. Future ideas might include a prospective enrollment of psoriasis patients, disease severity assessment using combined scores, such as PGAxBSA, modified PASI (mPASI), and psoriasis log-based area and severity index (PLASI), and a calculation of the FIB-4 cut-off value in the study population for discriminating between LR-SF and HR-SF. In this study, we used FIB-4 threshold values as reported by international consensus. A cut-off value determined in the study population might eliminate possible populational intervariability. 

Nevertheless, to our knowledge, this is the first study, to date, to assess the usefulness of blood-count-derived inflammatory markers in predicting liver fibrosis. Additionally, this is the first study reporting PWR as an inflammatory marker in liver fibrosis, and, as our results showed, it proves to be the most reliable one for discriminating liver fibrosis severity.

## 4. Materials and Methods

### 4.1. Study Population

We conducted a retrospective observational study that included patients diagnosed with psoriasis vulgaris in the Dermatology Department of Mures Clinical County Hospital, Romania, between January 2017 and December 2022. The inclusion criteria were patients older than 18 years of age, presenting for the first time in our department, diagnosed with psoriasis vulgaris in the aforementioned timeframe, and for whom data regarding disease severity and laboratory investigations were available. The following patients were excluded: patients diagnosed with other clinical forms of psoriasis, of pediatric age, for whom there were no available laboratory investigations or information regarding disease severity, patients with a known history of psoriatic arthritis, cardiovascular disease, liver diseases, malignant tumors, active infections, or diabetes, patients reporting daily alcohol use, and those who underwent 3 months of systemic treatment before enrollment with one of the following: steroids, classic immunosuppressive drugs (Methotrexate, Azathioprine, Cyclosporine), or innovative drugs (any type of biologics or PDE-4 inhibitors) were excluded.

### 4.2. Data Collection

The data was collected using the hospital’s electronic databases. For each patient, information regarding demographics (age, sex), clinical presentation, and laboratory parameters were extracted. Psoriasis severity was assessed using the Body Surface Area (BSA) score and defined as follows: mild (BSA < 5%), and moderate-to-severe (BSA > 10%). The following laboratory parameters were analyzed: complete white blood cell count (WBC), leucocyte subsets (neutrophils, lymphocytes, and monocytes) count, platelet count, alanine-aminotransferase (ALT), aspartate aminotransferase (AST), and gamma-glutamyl transferase (GGT) levels. For patients presenting multiple times in our department in the aforementioned time interval, data referring to the first presentation was taken into account.

### 4.3. Biomarkers

The following blood count-derived inflammatory markers were calculated: NLR, derived neutrophil-to-lymphocyte ratio (d-NLR), PLR, monocyte-to-lymphocyte ratio (MLR), SII, systemic inflammation response index (SIRI), aggregate index of systemic inflammation (AISI), and PWR. Liver fibrosis assessment was established based on the FIB-4 index. Additional non-invasive fibrosis markers, such as APRI, AAR, and GPR were evaluated. The formulas for the aforementioned markers are depicted in Table 6. 

### 4.4. Study Outcome

The primary endpoint of our study was to assess whether blood count-derived inflammatory markers may serve as predictors of liver fibrosis severity in patients with psoriasis vulgaris. Fibrosis severity was assessed using the FIB-4 index according to international consensus, and quantified as follows: mild fibrosis (low risk of significant fibrosis: LR-SF, F0-F1) if the FIB-4 index was lower than 1.3, and moderate-to-severe fibrosis (high risk of significant fibrosis: HR-SF, >F2) if FIB-4 was over 1.3 [9,53,54]. Second, we investigated whether non-invasive fibrosis markers such as APRI, AAR, and GPR were efficient in predicting liver fibrosis in patients with psoriasis vulgaris in comparison with the FIB-4 score. 

### 4.5. Statistical Analysis

The statistical analysis was performed using, and MedCalc Statistic software for Windows, version 22.014. Normality was tested using the Shapiro-Wilk test. Continuous variables were expressed as the median or mean and standard deviation, while for categorical variables, the absolute count (n) and proportions were given. Categorical variables were compared by Chi-square test, while the independent Mann-Whitney test was used for continuous variables. Correlations were evaluated by Spearman’s correlation coefficient. The performance of inflammatory scores for predicting liver fibrosis severity was assessed using receiver operating characteristic (ROC) curve analysis and the area under the ROC curves (AUCs). The optimal cut-off values for relevant systemic inflammatory markers were determined using the Youden Index from the ROC curve. The DeLong Z test was used to compare the AUCs of the serum models. Multivariate logistic regression adjusted for sex and age, with variables with *p* < 0.1 in univariate analysis, was performed to identify independent prognostic factors associated with liver fibrosis severity. The Hosmer-Lemeshow test was used to assess the goodness of fit for the logistic regression model. *p* < 0.05 was considered statistically significant throughout all the analyses.

## 5. Conclusions

In our study group, MLR, PWR, and AISI were identified as being prognostic factors useful for assessing liver fibrosis severity in psoriasis. Additionally, APRI and AAR may be used as additional non-invasive markers to assess liver fibrosis. These findings bring new information and highlight once more the strength between psoriasis, systemic inflammation, and associated comorbidities. 

Taking into account the ease and low cost of these ratios, they can be used for a quick and efficient patient risk assessment, guide future diagnostic means, and initial therapeutical decisions.

## Figures and Tables

**Table 1:** Clinical and laboratory characteristics of the study population.

Variables	All Patients	LR-SF (n = 246)	HR-SF (n = 113)	*p*-Value
Age	54.76 ± 16.36	50.07 ± 16.10	64.98 ± 11.63	<0.001
Gender				
Male	216 (60.17%)	141 (57.32%)	75 (66.37%)	0.100
Female	143 (39.83%)	105 (42.68%)	38 (33.63%)	
Disease severity				
Mild	177 (49.30%)	132 (53.66%)	45 (39.82%)	0.015
Moderate-severe	182 (50.70%)	114 (46.34%)	68 (60.18%)	
AST	19 [18–20.56]	16 [15–17]	31 [26–37]	<0.001
ALT	22 [21–24]	21 [19–22.86]	28 [23.61–33]	0.001
GGT	30 [27–32]	24.5 [20.14–28]	48 [36–68.40]	<0.001
Platelets	238.02 [228.90–243.00]	270.44 [261.67–279.21]	188.67 [178.59–198.75]	<0.001
WBC	7.50 [7.17–7.85]	7.73 [7.37–8.09]	6.84 [6.43–7.67]	0.001
Neutrophils	4.44 [4.23–4.75]	4.53 [4.28–4.88]	4.12 [3.77–4.66]	0.02
Lymphocytes	2.08 [1.97–2.23]	2.22 [2.08–2.30]	1.85 [1.67–2.00]	<0.001
Monocytes	0.50 [0.48–0.53]	0.49 [0.46–0.52]	0.53 [0.48–0.56]	0.33
PLR	115.19 [110.12–120.96]	117.49 [112.46–153.81]	102.51 [89.95–117.91]	<0.001
NLR	2.05 [1.91–2.21]	2.04 [1.82–2.23]	2.08 [1.95–2.32]	0.58
d-NLR	1.56 [1.44–1.66]	1.55 [1.42–1.67]	1.60 [1.44–1.73]	0.96
MLR	0.24 [0.22–0.25]	0.22 [0.21–0.23]	0.28 [0.26–0.30]	<0.001
ESR	15 [12.74–17.26]	14.26 [12.47–16.53]	17 [13.63–20]	0.07
SII	480.22 [453.86–524.81]	526.07 [480.15–570.06]	431.53 [387.45–462.05]	<0.001
SIRI	1.04 [0.94–1.11]	0.99 [0.90–1.11]	1.07 [0.98–1.28]	0.42
AISI	258.40 [231.92–274]	273.26 [248.12–285.65]	214.55 [187.50–250.25]	0.001
APRI	0.22 [0.20–0.23]	0.18 [0.16–0.19]	0.49 [0.41–0.55]	<0.001
AAR	0.88 [0.82–0.93]	0.75 [0.70–0.81]	1.10 [1.02–1.25]	<0.001
PWR	32.86 [30.99–33.98]	34.49 [22.05–36.53]	27.40 [25.39–31.12]	<0.001
GPR	0.12 [0.11–0.13]	0.09 [0.08–0.10]	0.24 [0.20–0.33]	<0.001

AST, aspartate aminotransferase; ALT, alanine aminotransferase; GGT, gamma-glutamyl transferase; WBC, white blood cell count; PLR, platelet-to-lymphocyte ratio; NLR, neutrophil-to-lymphocyte ratio; d-NLR, derived neutrophil-to-lymphocyte ratio; MLR, monocyte-to-lymphocyte ratio; ESR, erythrocyte sedimentation rate; SII, systemic immune inflammation index; SIRI, systemic inflammation response index; AISI, aggregate index of systemic inflammation; APRI, AST to platelet ratio; AAR, AST to ALT ratio; PWR, platelet-to-white blood cell ratio; GPR, GGT to platelet ratio index.

**Table 2:** Correlation between serological markers and liver fibrosis severity.

Marker	r	*p*-Value	Marker	r	*p*-Value
AST	0.49	<0.001	MLR	0.20	<0.001
ALT	0.21	<0.001	SII	−0.22	<0.001
GGT	0.39	<0.001	AISI	−0.17	<0.001
Platelets	−0.546	<0.001	PWR	−0.25	<0.001
WBC	−0.17	0.001	APRI	0.63	<0.001
Lymphocytes	−0.20	<0.001	AAR	0.44	<0.001
PLR	−0.19	<0.001	GPR	0.46	<0.001

AST, aspartate aminotransferase; ALT, alanine aminotransferase; GGT, gamma-glutamyl transferase; WBC, white blood cell count; PLR, platelet-to-lymphocyte ratio.

**Table 3:** Predictive performance of hepatic NITs.

	AUC (95% CI)	*p*-Value	Cut-Off	Se (%)	Sp (%)	Youden Index J	*p*-Value *
PLR	0.618 (0.565–0.668)	<0.001	94.68	46.90	76.42	0.23	0.19
MLR	0.624 (0.571–0.674)	<0.001	0.26	58.41	65.85	0.24	0.44
SII	0.640 (0.588–0.690)	<0.001	828.77	93.81	26.42	0.20	0.71
AISI	0.607 (0.555–0.658)	<0.001	273.09	66.37	50.00	0.16	0.33
PWR	0.657 (0.606–0.706)	<0.001	27.59	52.21	74.80	0.27	-

Se: sensitivity; Sp: specificity; * Compared to PWR; PLR, platelet-to-lymphocyte ratio; MLR, monocyte-to-lymphocyte ratio; MLR, monocyte-to-lymphocyte ratio; AISI, aggregate index of systemic inflammation; PWR, platelet-to-white blood cell ratio.

**Table 4:** Predictive performance of hepatic NITs.

	AUC (95% CI)	*p*-Value	Cut-Off	Se (%)	Sp (%)	Youden Index J	*p*-Value *
APRI	0.889 (0.852–0.920)	<0.001	0.22	91.15	69.11	0.60	-
AAR	0.774 (0.727–0.816)	<0.001	0.89	75.22	66.67	0.42	<0.001
GPR	0.786 (0.740–0.828)	<0.001	0.14	74.34	73.98	0.48	<0.001

Se: sensitivity; Sp: specificity; * Compared to APRI; APRI, AST to platelet ratio; AAR, AST to ALT ratio; GPR, GGT to platelet ratio index.

**Table 5:** Predictors of significant liver fibrosis in psoriasis patients.

Parameter	OR	95% CI	*p*-Value
**Demographic characteristics**			
Age > 50 years old	4.63	2.57–8.36	<0.001
Male sex	0.78	0.48–1.27	0.127
Moderate-severe psoriasis	1.70	1.06–2.73	0.028
**Inflammatory markers**			
PLR	1.02	0.99–1.06	0.097
MLR	3.51	1.69–7.29	<0.001
PWR	0.94	0.99–1.02	<0.001
SII	0.99	0.99–1.01	0.150
AISI	0.98	0.98–0.99	0.009
**Hepatic NITs**			
APRI	11.68	7.44–18.32	<0.001
AAR	13.26	5.37–32.78	<0.001
GPR	4.54	0.70–29.43	0.110

PLR, platelet-to-lymphocyte ratio; MLR, monocyte-to-lymphocyte ratio; PWR, platelet-to-white blood cell ratio; SII, systemic immune inflammation index; AISI, aggregate index of systemic inflammation; APRI, AST to platelet ratio; AAR, AST to ALT ratio; GPR, GGT to platelet ratio index.

**Table 6:** Formulas of blood-count-derived markers.

Marker	Formula
NLR	Neutrophil count/lymphocyte count [×10^3^/μL]
d-NLR	Neutrophil count/(WBC-neutrophil count) [×10^3^/μL]
PLR	Platelet count/lymphocyte count [×10^3^/μL]
MLR	Monocyte count/lymphocyte count [×10^3^/μL]
SII	(Neutrophil count × platelet count)/lymphocyte count [×10^3^/μL]
SIRI	(Neutrophil count × monocyte count)/lymphocyte count [×10^3^/μL]
AISI	(Neutrophil count × monocyte count × platelet count)/lymphocyte count [×10^3^/μL]
PWR	Platelet count/WBC [×10^3^/μL]
FIB-4	(Age [years] × AST [U/L])/(platelet count [×10^3^/μL] × √ALT [U/L])
APRI	[(AST/upper limit of the normal AST range) × 100]/platelet count [×10^3^/μL]
AAR	AST/ALT [U/L]
GPR	GGT [U/L]/platelet count [×10^3^/μL]

NLR, neutrophil-to-lymphocyte ratio; d-NLR, derived neutrophil-to-lymphocyte ratio; PLR, platelet-to-lymphocyte ratio; MLR, monocyte-to-lymphocyte ratio; SII, systemic immune inflammation index; SIRI, systemic inflammation response index; AISI, aggregate index of systemic inflammation; PWR, platelet-to-white blood cell ratio; FIB-4, Fibrosis-4 index; APRI, AST to platelet ratio; AAR, AST to ALT ratio; GPR, GGT to platelet ratio index.

## Data Availability

All data presented can be made available upon request.
